# Adrenomedullin: a marker of impaired hemodynamics, organ dysfunction, and poor prognosis in cardiogenic shock

**DOI:** 10.1186/s13613-016-0229-2

**Published:** 2017-01-04

**Authors:** Heli Tolppanen, Mercedes Rivas-Lasarte, Johan Lassus, Jordi Sans-Roselló, Oliver Hartmann, Matias Lindholm, Mattia Arrigo, Tuukka Tarvasmäki, Lars Köber, Holger Thiele, Kari Pulkki, Jindrich Spinar, John Parissis, Marek Banaszewski, Jose Silva-Cardoso, Valentina Carubelli, Alessandro Sionis, Veli-Pekka Harjola, Alexandre Mebazaa

**Affiliations:** 1 INSERM UMR-S942, Paris, France; 2 Heart Center, Päijät-Häme Central Hospital, Lahti, Finland; 3 Heart and Lung Center, Helsinki University and Helsinki University Hospital, Helsinki, Finland; 4 Intensive Cardiac Care Unit, Cardiology Department, Hospital de la Santa Creu i Sant Pau, Biomedical Research Institute IIB-SantPau, Universidad Autónoma de Barcelona, Barcelona, Spain; 5 Sphingotec GmbH, Hennigsdorf, Germany; 6 Department of Cardiology, Rigshospitalet, University of Copenhagen, Copenhagen, Denmark; 7 Department of Emergency Care, Helsinki University and Helsinki University Hospital, Helsinki, Finland; 8 Medical Clinic II, University Hospital Schleswig-Holstein, University Heart Center Lübeck, Lübeck, Germany; 9 Department of Clinical Chemistry, University of Eastern Finland, Kuopio, Finland; 10 Eastern Finland Laboratory Centre, Kuopio, Finland; 11 Department of Internal Medicine and Cardiology, University Hospital Brno, Brno, Czech Republic; 12 International Clinical Research Centre (ICRC), Brno, Czech Republic; 13 Heart Failure Clinic and Secondary Cardiology Department, Attikon University Hospital, Athens, Greece; 14 Intensive Cardiac Therapy Clinic, Institute of Cardiology, Warsaw, Poland; 15 Department of Cardiology, CINTESIS, Porto Medical School, São João Hospital Center, University of Porto, Porto, Portugal; 16 Division of Cardiology, Department of Medical and Surgical Specialties Radiological Sciences and Public Health, University and Civil Hospital of Brescia, Brescia, Italy; 17 Department of Anesthesia and Critical Care, University Hospital Saint Louis Lariboisière, APHP, Paris, France; 18 University Paris Diderot, Sorbonne Paris Cité, Paris, France; 19 Department of Cardiology, University Heart Center, 8091 Zürich, Switzerland; 20 Department of Cardiology, University Hospital Zürich, 8091 Zürich, Switzerland

**Keywords:** Adrenomedullin, Cardiogenic shock, Biomarkers, Lactate, Hemodynamics, Mortality

## Abstract

**Background:**

The clinical CardShock risk score, including baseline lactate levels, was recently shown to facilitate risk stratification in patients with cardiogenic shock (CS). As based on baseline parameters, however, it may not reflect the change in mortality risk in response to initial therapies. Adrenomedullin is a prognostic biomarker in several cardiovascular diseases and was recently shown to associate with hemodynamic instability in patients with septic shock. The aim of our study was to evaluate the prognostic value and association with hemodynamic parameters of bioactive adrenomedullin (bio-ADM) in patients with CS.

**Methods:**

CardShock was a prospective, observational, European multinational cohort study of CS. In this sub-analysis, serial plasma bio-ADM and arterial blood lactate measurements were collected from 178 patients during the first 10 days after detection of CS.

**Results:**

Both bio-ADM and lactate were higher in 90-day non-survivors compared to survivors at all time points (*P* < 0.05 for all). Lactate showed good prognostic value during the initial 24 h (AUC 0.78 at admission and 0.76 at 24 h). Subsequently, lactate returned normal (≤2 mmol/L) in most patients regardless of later outcome with lower prognostic value. By contrast, bio-ADM showed increasing prognostic value from 48 h and beyond (AUC 0.71 at 48 h and 0.80 at 5–10 days). Serial measurements of either bio-ADM or lactate were independent of and provided added value to CardShock risk score (*P* < 0.001 for both). Ninety-day mortality was more than double higher in patients with high levels of bio-ADM (>55.7 pg/mL) at 48 h compared to those with low bio-ADM levels (49.1 vs. 22.6%, *P* = 0.001). High levels of bio-ADM were associated with impaired cardiac index, mean arterial pressure, central venous pressure, and systolic pulmonary artery pressure during the study period. Furthermore, high levels of bio-ADM at 48 to 96 h were related to persistently impaired cardiac and end-organ function.

**Conclusions:**

Bio-ADM is a valuable prognosticator and marker of impaired hemodynamics in CS patients. High levels of bio-ADM may show shock refractoriness and developing end-organ dysfunction and thus help to guide therapeutic approach in patients with CS.

*Study identifier of CardShock study* NCT01374867 at clinicaltrials.gov

**Electronic supplementary material:**

The online version of this article (doi:10.1186/s13613-016-0229-2) contains supplementary material, which is available to authorized users.

## Background

Cardiogenic shock (CS) is a state of global tissue hypoperfusion caused by severe cardiac dysfunction. In spite of advances in therapeutic options, the short-term mortality associated with CS remains unacceptably high [1, 2]. While very early mortality is largely related to sudden and severe circulatory failure, subsequent death is strongly influenced by activation of neurohumoral and inflammatory responses leading to multiorgan failure [3]. Risk stratification is crucial in order to accurately identify patients that could potentially benefit from more aggressive strategies, and moreover, to identify advanced stages of shock when restoring cardiac function may not reverse end-organ failure. The CardShock risk score was recently introduced to help risk stratification in the early phase of CS. The score includes lactate levels and six other clinical variables available at the time of detection of shock [2]. However, as based on baseline parameters, the score may not reflect the change in mortality risk in response to initial therapies.

At present, few biomarkers have been proven beneficial in risk stratification of patients with CS. Lactate is an established marker of hemodynamic instability and prognosis in critically ill patients [4–6]. Adrenomedullin (ADM) has been shown as prognosticator in CS after an acute coronary syndrome [7]. The study was small, monocentric, and based only on one sample measured at 24 h, however. In patients with septic shock, higher ADM levels were associated with hemodynamic instability, requirement of vasopressor therapy, and increased mortality [8]. The aim of the present study was to evaluate the prognostic value and association with hemodynamic parameters of serial measurements of mature bioactive ADM (bio-ADM) in patients with CS, in order to help risk assessment and support clinical decision in CS.

## Methods

### Study population and endpoints

CardShock study (NCT01374867) is a prospective European multicenter and multinational cohort study that enrolled consecutive CS patients in 9 centers in 8 countries between October 2010 and December 2012. The inclusion criteria were systolic blood pressure <90 mmHg for 30 min despite fluid administration or need for vasoactive therapy, and one or more signs of organ hypoperfusion (cool extremities, confusion or altered mental status, oliguria <0.5 ml/kg/h for the previous 6 h, or blood lactate >2 mmol/l), cardiac origin of the state of hypoperfusion, and age over 18 years. Study inclusion was within the first 6 h of the detection of shock. Exclusion criteria were shock caused by ongoing hemodynamically significant arrhythmias and shock after cardiac or non-cardiac surgery. For this sub-study, 178 patients with biomarker data (lactate and bio-ADM) available were included.

The primary endpoint of the study was to determine the prognostic value of serial measurements of bio-ADM on mortality prediction at 90 days. Secondary endpoint was to describe the relationship of bio-ADM and lactate with hemodynamic parameters.

### Study protocol

Detailed medical history and patient characteristics were collected. Clinical signs with routine laboratory measurements, including lactate which was measured locally, were registered at presentation to the hospital. A total of 69 (39%) patients had pulmonary artery catheter, and additional 42 (24%) patients had central venous pressure monitoring. As per study protocol, all patients had echocardiography performed at baseline and at 72 h. Patients were treated according to local practice in each hospital. Vital status during follow-up was determined through direct contact with the patient or next of kin, or through population and hospital registries. Three patients were lost to follow-up; in the mortality analyses their cases were censored at the time of hospital discharge. Serial plasma samples were taken at various time points after presentation and immediately frozen and stored at −80 °C. Both bio-ADM and arterial lactate were measured at 0, 12, 24, 48, 72, and 96 h, and bio-ADM again at 5–10 days.

The CardShock study was approved by local ethics committees at the participating centers and conducted in accordance with the Declaration of Helsinki. All patients or their next of kin gave informed consent.

### Bio-ADM measurement

All bio-ADM measurements were taken blinded for clinical data in the laboratories of Sphingotec GmbH, Hennigsdorf, Germany, with a previously described immunoassay [8]. Mid-regional pro-Adrenomedullin (MR-proADM), a non-bioactive precursor of ADM, has been used in recent years to overcome the obstacles of mature ADM measurement relating to analyte stability and interference with complement factor H in the measurement [9–11]. In our study, the novel immunoassay allowed reliable ultrasensitive measurement of bioactive ADM peptide from small sample volume (50 uL of plasma), contrary to the earlier measurement of mature ADM levels [12]. Briefly, a one-step sandwich-coated tube chemiluminescence immunoassay was used based on acridinium NHS-ester labeling for the detection of human ADM in plasma. More detailed description of the bio-ADM measurement is provided in Additional file 1. The upper limit of normal values of bio-ADM with the assay used is 43 pg/mL [8].

### Statistical analysis

Results are presented as numbers (*n*) and percentages (%), and means with standard deviations (SD) or medians with interquartile ranges (IQRs) as appropriate. Between groups, comparisons were made using Chi-square test, *t* test, or Wilcoxon rank-sum test as appropriate.

Cox proportional hazards regression was used to analyze the time-dependent effect of serial measurements of bio-ADM and lactate on 90-day survival in uni- and multivariable analyses [13, 14]. Hazard ratios (HRs) are given with 95% confidential intervals (CIs). Both biomarkers were tested for independency from the previously developed CardShock risk score [2], which summarizes seven clinical parameters, which were associated with in-hospital mortality. The model included baseline lactate as its strongest component, as well as age over 75 years, acute coronary syndrome as the etiology of CS, previous history of myocardial infarction or coronary artery bypass surgery, altered mental status at presentation, renal function, and left ventricular ejection fraction below 40% at baseline. The assumptions of proportional hazard were tested for all variables. For all analyses, biomarkers (bio-ADM and lactate) were log-transformed and HR was standardized to describe the HR for a biomarker change in one IQR. Wald statistics were used to investigate the prognostic value of each biomarker and their combination when measured at each time point. To give an effect measure for the prognostic value of bio-ADM and lactate in 90-day mortality, the receiver-operating characteristic (ROC) curve analysis was performed and areas under ROC curves (AUCs) were calculated. Kaplan–Meier curves were also used in survival analyses. Dichotomization of patients was based on bio-ADM level 55.7 mg/ml, which was the optimal cutoff with highest sensitivity and specificity for 90-day mortality when measured at 48 h, and similar to the median values of bio-ADM during the first 96 h (range of medians at 0–96 h 54.5–59.9 pg/ml).

For comparison of biomarker levels with hemodynamic parameters, median of all biomarker measurements taken during the initial 96 h of each patient was used. Dichotomization was based on bio-ADM level of 55.7 pg/mL and lactate level of 1.63 mmol/L, which was the median value of each patient’s median lactate level during the first 96 h. For comparison of hemodynamic measures and end-organ dysfunction at 48–96 h, the median value of the measures between 48 and 96 h of each patient was used, and dichotomization was based on median value of bio-ADM at 48–96 h with the cutoff level of 55.7 pg/mL. A two-sided *P* value <0.05 was regarded as statistically significant. The statistical analyses were performed using R version 2.5.1 (http://www.r-project.org, library Design, Hmisc, ROCR), SPSS 21.0 statistical software (IBM Corp, Armonk, NY, USA) and STATA (version 13, Statacorp, Texas, USA).

## Results

The mean age of the 178 patients included in this study was 66 ± 12 years, and 137 (74%) were men. Most common etiology of CS was acute coronary syndrome (78%). The overall 90-day mortality was 43% (*n* = 75). Table 1 describes the patient characteristics of the 90-day survivors and non-survivors. Twenty-nine (16%) patients died before 48 h from the detection of shock, and the remaining 46 (26%) patients died between 48 h and 90 days. The earlier deaths tended to occur more often due to myocardial infarction (71 vs. 51%, *P* = 0.086) and less often due to worsening heart failure (17 vs. 42%, *P* = 0.017). On the contrary, the later occurring deaths were numerically more often related to infection, renal failure, and stroke, although these differences did not reach statistical significance (Additional file 2: Table S1).

**Table 1:** Characteristics of 90-day survivors and non-survivors

	Survivors (*n* = 103)	Non-survivors (*n* = 75)	*P* value
Age	63 (13)	71 (11)	<0.001
Male gender	82 (80%)	51 (68%)	0.08
CardShock risk score	3.4 (1.7)	5.5 (1.5)	<0.001
Medical history			
Hypertension	59 (57%)	51 (68%)	0.15
Hyperlipidemia	41 (40%)	44 (59%)	0.013
Diabetes	23 (22%)	30 (40%)	0.011
Smoker	48 (47%)	23 (31%)	0.032
Ischemic heart disease	23 (22%)	36 (48%)	<0.001
Previous infarction	16 (16%)	29 (39%)	<0.001
Previous CABG	1 (1%)	10 (13%)	0.001
Chronic heart failure	13 (13%)	16 (21%)	0.12
Stroke or TIA	8 (8%)	8 (11%)	0.5
Peripheral artery disease	6 (6%)	13 (17%)	0.014
Asthma or COPD	11 (11%)	9 (12%)	0.8
Status at inclusion			
Altered mental status	56 (55%)	61 (81%)	<0.001
Systolic BP, mmHg	80 (70–85)	75 (66–80)	0.016
Mean BP, mmHg	58 (53–64)	53 (47–60)	0.011
Heart rate	88 (27)	89 (31)	0.94
LVEF, %	36 (15)	29 (12)	<0.001
hs-TnT, ng/L	1366 (183–4191)	2862 (1124–7842)	0.008
NT-proBNP, ng/L	2026 (443–7101)	5174 (1447–16,547)	0.001
eGFR, ml/min/1.72m2	71 (29)	51 (27)	<0.001
Post-resuscitation	20 (19%)	27 (36%)	0.013
Etiology of cardiogenic shock			
Acute coronary syndrome	77 (75%)	65 (87%)	0.051
Left main stenosis	11 (14%)	15 (25%)	0.09
Three-vessel disease	17 (22%)	25 (42%)	0.008
PCI	66 (88%)	52 (80%)	0.2
Thrombolysis	12 (16%)	5 (8%)	0.13
Myocarditis or Takotsubo	8 (8%)	0 (0%)	
Valvular cause	6 (6%)	6 (8%)	0.6
Chronic cardiomyopathy/heart failure	11 (11%)	4 (5%)	0.2
In-hospital management			
Any inotrope	74 (76%)	54 (81%)	0.5
Any vasopressor	75 (76%)	64 (94%)	0.002
Invasive ventilation	54 (52%)	56 (75%)	0.003
IABP treatment	51 (50%)	45 (60%)	0.17
ECMO	2 (2%)	1 (1%)	0.8
LVAD	1 (1%)	4 (6%)	0.08

Continuous variables expressed as mean (standard deviation) or median (interquartile range), as appropriate; categorical variables expressed as number (percentage). *BMI* body mass index, *CABG* coronary artery bypass graft surgery, *COPD* chronic obstructive pulmonary disease, *TIA* transient ischemic attack, *BP* blood pressure, *LVEF* left ventricular ejection fraction, *hs-TnT* high sensitive troponin T, *NT-proBNP* N-terminal pro-brain natriuretic peptide, *eGFR* estimated glomerular filtration rate, *PCI* percutaneous coronary intervention, *IABP* intra-aortic balloon pump, *LVAD* left ventricular assist device, *ECMO* extracorporeal membrane oxygenation

### Bio-ADM and lactate levels in survivors and non-survivors

**Fig. 1:**
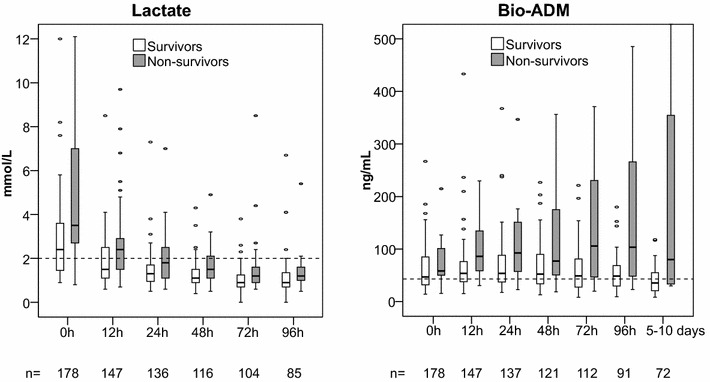
Time course of lactate (*left*) and bioactive adrenomedullin (bio-ADM) (*right*) levels in 90-day survivors (*white boxes*) and non-survivors (*gray boxes*). *Box* and *whisker plot*: central line = median, box = interquartile range, whiskers = 5th and 95th percentile, circles = outliers. *Scatted line* upper normal limit (2 mmol/L for lactate and 43 pg/mL for bio-ADM [8]). *n* = number of samples at each study time point. *P* values for all comparisons between survivors and non-survivors at each time point were <0.001, except for lactate at 48, 72, and 96 h, and bio-ADM at 0 h with *P* < 0.05

Plasma bio-ADM levels and arterial blood lactate were higher in non-survivors compared to survivors at all time points. The highest lactate levels were observed at baseline both in survivors and non-survivors (2.2 and 5.0 mmol/L, respectively, *P* < 0.0001). The median levels of lactate returned to normal values within 12 h in survivors and within 24 h in non-survivors (Fig. 1). Hence, at 24 h 76% of all patients had normal lactate levels. The time course of plasma bio-ADM levels was divergent between survivors and non-survivors; bio-ADM levels stayed close to the upper normal limit (43 pg/mL) in survivors while remained elevated in non-survivors (Fig. 1).

### Prognostic value of bio-ADM and lactate levels

Serial measurement of the biomarkers showed that for both bio-ADM and lactate, a normalization of concentration was associated with a decrease in mortality risk, while a continuing high concentration or increasing concentrations were associated with a high mortality risk. In time-dependent Cox model, serial bio-ADM and lactate measures were associated with increased 90-day risk of death in univariate time-dependent Cox analysis (HR 2.22, CI 1.76–2.80, *P* < 0.001 and HR 3.83, CI 2.73–5.37, *P* < 0.001, respectively) and after adjustment for the CardShock risk score (HR 1.62, 95% CI 1.26–2.09, *P* < 0.001, and HR 2.78, 95% CI 1.94–3.97, *P* < 0.001, respectively). Time-dependent Cox model for serial bio-ADM and lactate was associated with increased risk of 90-day mortality also when selecting only patients with CS caused by ACS (HR 1.49, CI 1.10–2.02, *P* = 0.01 for bio-ADM and HR 2.76, CI 1.94–3.92, *P* < 0.001 for lactate).

**Fig. 2:**
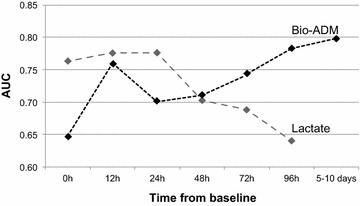
Area under the curve (AUC) of lactate (*gray line*) and bioactive adrenomedullin, bio-ADM, (*black line*) to discriminate between 90-day survivors and non-survivors at each time point

**Fig. 3:**
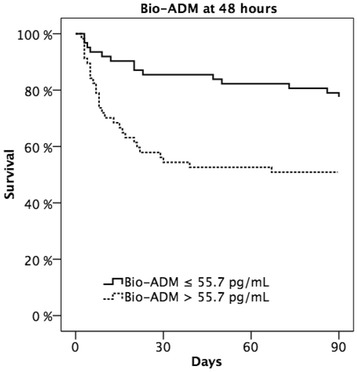
Ninety-day survival of patients with high (>55.7 pg/mL) or low (≤55.7 pg/mL) levels of bio-ADM at 48 h (*P* value = 0.001 with log-rank testing)

In the early phase of CS, lactate had good prognostic value (AUC at baseline 0.76, 95% CI 0.69–0.82) that rapidly decreased, whereas bio-ADM had incremental prognostic value with an AUC of 0.71 at 48 h (95% CI 0.62–0.79) up to 0.80 (95% CI 0.78–0.91) at 5–10 days (Fig. 2). The 90-day mortality was more than double higher in patients with high levels of bio-ADM at 48 h compared to those with low levels of bio-ADM (mortality 49.1 vs. 22.6%, *P* = 0.001), as shown in Fig. 3.

A more in-depth analysis of individual time points revealed that for lactate, its measurement at early time points provided added value to risk prediction, and later time points showed poor prognostic ability. For bio-ADM, the later time points provided the most added value and best discriminatory power (Table 2).

**Table 2:** Predictive value for 90-day mortality with Wald statistics of lactate and bio-ADM at each time point after the detection of shock

	*x* ^2^	*P* value
0 h		
Lactate	38.44	<0.0001
Bio-ADM	0.09	0.8
Total	42.76	<0.0001
12 h		
Lactate	23.99	<0.0001
Bio-ADM	4.28	0.039
Total	46.69	<0.0001
24 h		
Lactate	38.03	<0.0001
Bio-ADM	2.41	0.12
Total	44.82	<0.0001
48 h		
Lactate	3.14	0.077
Bio-ADM	9.79	0.002
Total	20.58	<0.0001
72 h		
Lactate	2.52	0.11
Bio-ADM	8.01	0.0047
Total	15.08	0.0005
96 h		
Lactate	0.18	0.7
Bio-ADM	17.01	<0.0001
Total	19.95	<0.0001

Lactate and bio-ADM levels were log10-transformed for the analysis. During the first 24 h, only lactate contributes to mortality prediction, later only bio-ADM contributes to prediction

### Bio-ADM and hemodynamic alterations

**Fig. 4:**
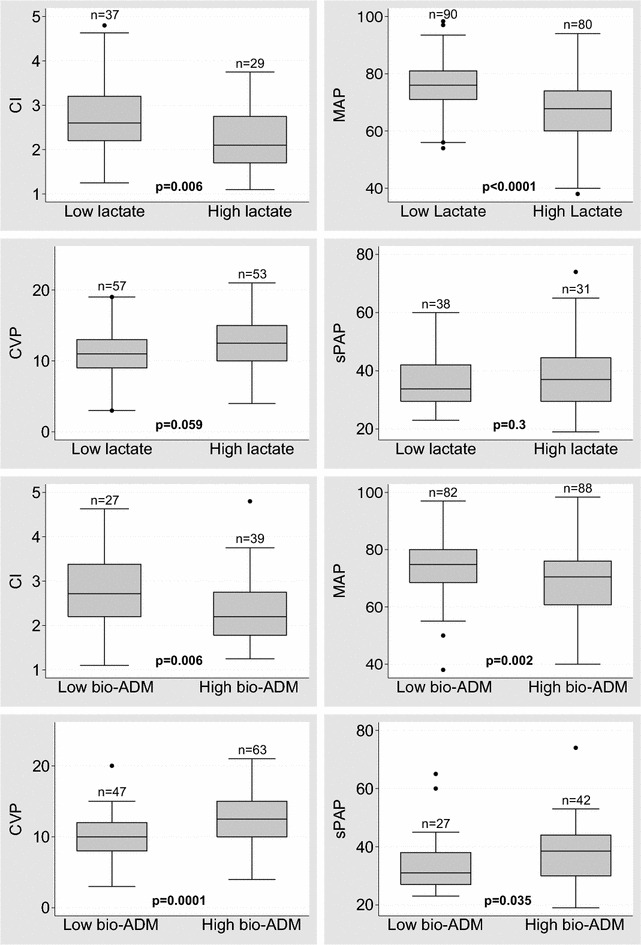
Comparison of hemodynamics between patients with **a** high and low bioactive adrenomedullin (bio-ADM) and **b** high and low lactate levels. Data presented as median with interquartile range of hemodynamic and biomarker data collected during the initial 96 h. The number (*n*) of patients with each measure is indicated on top of the *box plot*. *CI* cardiac index (L/min/m2), *MAP* mean arterial pressure (mmHg), *CVP* central venous pressure (mmHg), *sPAP* systolic pulmonary artery pressure (mmHg). Dichotomization of biomarker values was based on median lactate (1.63 mmol/L) or bio-ADM (55.7 pg/ml) during the study period. *P* value based on Wilcoxon rank-sum test

**Fig. 5:**
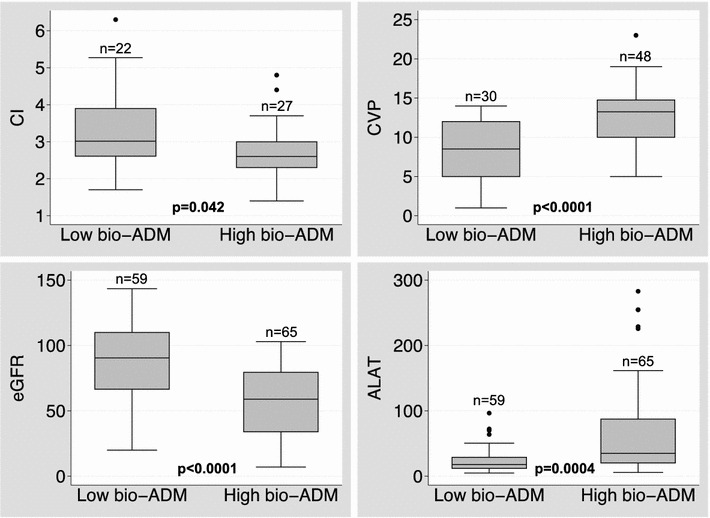
Association of bioactive adrenomedullin (bio-ADM) with hemodynamics and organ dysfunction at 48–96 h. Dichotomization of bio-ADM was based on 55.7 pg/ml cut-point at 48–96 h. The number (*n*) of patients with each measure is indicated on top of the box plot. *P* value based on Wilcoxon rank-sum test. *CI* cardiac index (L/min/m2), *CVP* central venous pressure (mmHg), *eGRF* estimated glomerular filtration rate (ml/min/1.73m2), *ALAT* alanine transaminase (UI/L)

Overall, both high bio-ADM levels and high lactate levels during the study period were associated with low cardiac index and low mean arterial pressure. In addition, high bio-ADM levels, but not high lactate levels, were associated with high central venous pressure and high systolic pulmonary artery pressure (Fig. 4). Furthermore, high bio-ADM levels at 48–96 h were associated with impaired cardiac and end-organ dysfunction, as shown in Fig. 5. Of note, at that time period, of the hemodynamic parameters only cardiac index was a good prognosticator of later outcome (Additional file 3: Table S2).

## Discussion

The present study shows that bio-ADM has strong prognostic value in CS when measured after the initial phase of management (at 48 h or later), and is associated with impaired hemodynamics and persistently impaired cardiac and end-organ dysfunction.

Lactate is a well-known marker of hemodynamic instability and disease severity in patients with shock. It is a marker of poor outcome if measured during the initial 24 h of intensive care unit admission in CS [5] or in overall critically ill patient population [6]. Our study confirmed the prognostic value of lactate during the first 24 h in CS. Later, in patients surviving the early phase of shock, arterial blood lactate levels returned normal in the majority of patients regardless of outcome, and the association with mortality was less significant.

In recent years, mature ADM, a hormone with potent vasodilatory and inotropic properties, or its precursor protein MR-proADM as its surrogate, have evolved as powerful prognostic markers in patients presenting with acute chest pain [15], dyspnea [16–18] and in those with acute heart failure [19]. In patients with acute myocardial infarction, high ADM levels have been associated with impaired left ventricular function and death [7, 20, 21]. Moreover, in patients with refractory CS requiring extracorporeal membrane oxygenation (ECMO) support, the levels of MR-proADM were found steadily elevated during first seven days and did not differ regardless of weaning success [22]. In our study, using a novel ultrasensitive method for bio-ADM measurement [8, 12], we showed that bio-ADM had good prognostic value in patients with CS. Indeed, bio-ADM was elevated during the whole study period in non-survivors, and high levels of bio-ADM were associated with increased short-term death, especially after 48 h, time when lactate had lower prognostic value than at baseline.

The exact source and the role of bio-ADM in CS are unknown. Plasma ADM is mainly derived from vascular endothelial cells, smooth muscle cells, and adventitial fibroblasts. Catecholamines, angiotensin II, and aldosterone, all of which are highly elevated in CS, are potent stimulators of ADM production [23]. Inflammatory cytokines, such as interleukins and TNFα, appearing in CS complicated by systemic inflammatory response syndrome [3], have also been advocated to stimulate ADM secretion [24–26]. Furthermore, in septic shock, high ADM levels are associated with decreased vascular tone and requirement of vasopressor therapy [8, 27]. In this study, we found that in contrast to lactate, bio-ADM levels are not only related to hypoperfusion (low cardiac index and low mean arterial pressure) but also with high cardiac filling pressures (central venous pressure and pulmonary artery pressure). We hypothesize that myocardial stunning is responsible for the activation of neurohumoral response (catecholamines, angiotensin II, interleukins), leading to bio-ADM production. Bio-ADM with its potent vasodilatory properties may act perpetuating shock and contributing to end-organ damage associated with poor prognosis. The exact pathways implicated in these processes need further investigation.

Interestingly, causes of death differed between patients who died before 48 h and later. The early deaths tended to occur more often due to myocardial infarction and less often due to worsening heart failure. Initial management in CS, as it was recently published in international recommendations [28–31], includes stabilization with volume expansion, inotropes, and vasopressors. This aggressive resuscitation in patients surviving the initial phase may be enough to reestablish a correct perfusion allowing lactate levels to decrease and even to normalize, as we found in our work. Nevertheless, in patients with activations of systemic inflammatory response, mortality has been reported to remain high related to other causes of death [32]. After initial medical stabilization, in many centers, mechanical assist devices are an increasingly used alternative to support circulation and allow recovery of stunned or hibernating myocardium if clinical signs of recovery are absent [33–35]. It seems that to increase survival, these advanced therapies should be started before irreversible end-organ dysfunction has occurred to carefully selected patients, considering the costs and possible complications of these therapies [36]. High levels of bio-ADM at 48 h or later may reflect a state of refractory shock with end-organ damage, despite normalization of lactate levels, and may help the clinician in a more accurate patient selection for advanced therapies, or guide in the difficult process of limiting the therapeutic effort.

Our study carries several limitations. Plasma samples were not available in all patients and at all time points. The high early mortality further decreased the number of subsequent samples. Nevertheless, considering the difficulties in prospectively studying patients with CS with timely plasma sampling, this is one of the largest cohorts of biomarker studies in patients with CS. As we used a novel technique for the identification of plasma bio-ADM, the levels of bio-ADM were directly comparable with only a few studies. However, the novel technique used is accurate and allows measures from small amounts of plasma and thus has the potential to become the technique of choice to assess ADM pathway in the future. Being still in experimental use, the measurement of bio-ADM is currently less available and a considerably more expensive laboratory test compared to lactate. Nevertheless, it is expected to become available on widespread fully automated platforms in near future. As this was an observational multinational study, the management of patients was not guided per protocol. Management in this study, however, reflects the real-world practice in European tertiary care university hospitals with high rate of accordance to the international recommendations. There were only a few patients treated with circulatory assist devices, thus preventing sub-analyses of these patients.

## Conclusions

As a conclusion, our study has a potentially important clinical implication, suggesting that bio-ADM measurement could be added to CS evaluation because of its prognostic value after the initial phase of management in patients with CS. Elevated levels of bio-ADM seem to be related to persistent cardiac and end-organ dysfunction and may support clinical decision when choosing therapeutic approach in patients with refractory CS. Whether risk estimation based on bio-ADM levels may help to optimize therapies and improve outcome needs further investigation.

## Additional files

**Additional file 1.** Expanded methods: bioactive adrenomedullin measurement.

**Additional file 2: Table S1.** Causes of death as reported by local investigators in patients who died early (within 48 h from the detection of shock) and late (more than 48 h from the detection of shock).

**Additional file 3: Table S2.** Area under curve (AUC) for predicting 90-day mortality of bioactive adrenomedullin (bio-ADM), lactate, mean arterial pressure (MAP), heart rate (HR), cardiac index (CI), and central venous pressure (CVP) measured at 48–96 h of the detection of cardiogenic shock.

## Abbreviations

CS: cardiogenic shock
ADM: adrenomedullin
bio-ADM: bioactive adrenomedullin
MR-proADM: mid-regional pro-adrenomedullin
SD: standard deviation
IQR: interquartile range
HR: hazard ratio
CI: confidential interval
LR: likelihood ratio
ROC: receiver-operating characteristics
AUC: area under curve
ECMO: extracorporeal membrane oxygenation
