# Supplementary material for: Adrenomedullin: a marker of impaired hemodynamics, organ dysfunction, and poor prognosis in cardiogenic shock
Source: Ann Intensive Care. 2017 Jan 4;7:6. doi: 10.1186/s13613-016-0229-2 (PMC5209311; doi:10.1186/s13613-016-0229-2)
Supplement: Supplementary file 1 — Additional file 1. Expanded methods: bioactive adrenomedullin measurement. [file 13613_2016_229_MOESM1_ESM.docx]

Additional file 1 – expanded methods

*Bio-ADM Measurement*

All bioactive adrenomedullin (bio-ADM) measurements were performed blinded in the laboratories of Sphingotec GmbH, Hennigsdorf, Germany, with a previously described immunoassay (1). Briefly, a one-step sandwich-coated tube chemiluminescence immunoassay was used based on Acridinium NHS-ester labeling for the detection of human ADM in plasma.

Mouse monoclonal antibodies (AB) directed against the mid region as solid phase and a labeled mouse monoclonal AB directed against the amidated C-terminal moiety of adrenomedullin are used in the assay. To label the anti-C-terminal antibodies (1 g/L) an incubation with MACN-acridinium-NHS (N-hydroxysuccinimide)-ester (1 g/L; InVent GmbH, Hennigsdorf, Germany) in a 1:5 molar ratio for 20 minutes at 22°C were performed and stopped by addition of 1/5 volume of 50 mmol/L glycine for 10 minutes at 22°C. As described before, the labeled antibody was diluted into assay buffer (300 mmol/L K-phosphate; 100 mmol/L NaCl; 10 mmol/L sodium EDTA; 5 g/L BSA (protease-free) (Sigma); 1 g/L each of nonspecific bovine and mouse IgG; 0.9 g/L Na-azide; 20 tabs/L Protease Inhibitor Cocktail (Roche Diagnostics GmbH, Penzberg, Germany); 10 μmol/L Amastatin; 20 μmol/L Leupeptin; pH 7.0). Dilutions of full-length human ADM peptide (American Peptide Company, Sunnyvale, CA, USA) in Calibrator Dilution Buffer (10 mmol/L Tris; 250 mmol/L NaCl; 2 g/L Triton X-100; 50 g/L BSA (protease-free); 20 tabs/L Protease Inhibitor Cocktail (Roche AG); pH 7.0) served as calibrators (1).

The immunoassay was performed with 50 μL of plasma samples or calibrators and 200 μL of the labeled detection antibody (800,000 relative light units (RLU) per 200 μL). Both were added to the coated tubes, incubated for 18 hours at 4°C and washed five times with wash solution (1 mL each). Finally, the chemiluminescence was measured for 1 s using the LB953 Multi-Tube Luminometer (BERTHOLD TECHNOLOGIES GmbH & Co. KG, Bad Wildbad, Germany). The analytical assay sensitivity was 2 pg/mL.

Mid-regional pro-adrenomedullin (MR-proADM) is generated in stoichiometric amounts to C-terminally glycine-extended adrenomedullin, which is not bioactive. ADM becomes bioactive only after conversion to a C-terminally amidated form. This conversion does not occur quantitatively and conversion rates differ depending on disease states. MR-proADM has been used in recent years to overcome the obstacles of mature adrenomedullin measurement relating to analyte stability and interference with complement factor H in the measurement (2-4). By careful selection of high affinity monoclonal antibodies against specific epitopes for use in the novel immunoassay used in our study the reliable ultrasensitive measurement of bio-ADM peptide from small sample volume (50 uL of plasma) has been achieved, contrary to the earlier measurement of mature adrenomedullin levels (5).

**References**

1. Marino R, Struck J, Maisel AS, Magrini L, Bergmann A, Di Somma S. Plasma adrenomedullin is associated with short-term mortality and vasopressor requirement in patients admitted with sepsis. Crit Care. 2014;18(1):R34. PubMed PMID: 24533868. Pubmed Central PMCID: PMC4056312.

2. Pio R, Martinez A, Unsworth EJ, Kowalak JA, Bengoechea JA, Zipfel PF, et al. Complement factor H is a serum-binding protein for adrenomedullin, and the resulting complex modulates the bioactivities of both partners. J Biol Chem. 2001 Apr 13;276(15):12292-300. PubMed PMID: 11116141.

3. Lewis LK, Smith MW, Yandle TG, Richards AM, Nicholls MG. Adrenomedullin(1-52) measured in human plasma by radioimmunoassay: plasma concentration, adsorption, and storage. Clinical chemistry. 1998 Mar;44(3):571-7. PubMed PMID: 9510864.

4. Struck J, Tao C, Morgenthaler NG, Bergmann A. Identification of an Adrenomedullin precursor fragment in plasma of sepsis patients. Peptides. 2004 Aug;25(8):1369-72. PubMed PMID: 15350706.

5. Di Somma S, Magrini L, Travaglino F, Lalle I, Fiotti N, Cervellin G, et al. Opinion paper on innovative approach of biomarkers for infectious diseases and sepsis management in the emergency department. Clin Chem Lab Med. 2013 Jun;51(6):1167-75. PubMed PMID: 23392907.
