# Supplementary material for: Adrenomedullin: a marker of impaired hemodynamics, organ dysfunction, and poor prognosis in cardiogenic shock
Source: Ann Intensive Care. 2017 Jan 4;7:6. doi: 10.1186/s13613-016-0229-2 (PMC5209311; doi:10.1186/s13613-016-0229-2)
Supplement: Supplementary file 2 — Additional file 2: Table S1. Causes of death as reported by local investigators in patients who died early (within 48 h from the detection of shock) and late (more than 48 h from the detection of shock). [file 13613_2016_229_MOESM2_ESM.docx]

|  | Died early (n=29) | Died late (n=46) | P-values (chi-square) |
| --- | --- | --- | --- |
| Myocardial infarction | 20 (71%) | 23 (51%) | 0.086 |
| Worsening heart failure | 5 (17%) | 19 (44%) | 0.017 |
| Pulmonary embolism | 1 (4%) | 0 | 0.2 |
| Wittnessed arrhytmic (VT/VF) sudden death | 1 (4%) | 1 (2%) | 0.8 |
| Stroke | 0 | 3 (7%) | 0.15 |
| Infection | 0 | 8 (19%) | 0.14 |
| Renal failure | 0 | 8 (19%) | 0.14 |
| Other cause | 2 (8%) | 9 (23%) | 0.13 |

Table S1. Causes of death as reported by local investigators in patients who died early (within 48 hours from the detection of shock) and late (more than 48 hours from the detection of shock).
