# Supplementary material for: Adrenomedullin: a marker of impaired hemodynamics, organ dysfunction, and poor prognosis in cardiogenic shock
Source: Ann Intensive Care. 2017 Jan 4;7:6. doi: 10.1186/s13613-016-0229-2 (PMC5209311; doi:10.1186/s13613-016-0229-2)
Supplement: Supplementary file 3 — Additional file 3: Table S2. Area under curve (AUC) for predicting 90-day mortality of bioactive adrenomedullin (bio-ADM), lactate, mean arterial pressure (MAP), heart rate (HR), cardiac index (CI), and central venous pressure (CVP) measured at 48–96 h of the detection of cardiogenic shock. [file 13613_2016_229_MOESM3_ESM.docx]

| **AUC for 90-day mortality** | | | |
| --- | --- | --- | --- |
|  | **48h** | **72h** | **96h** |
| **Bio-ADM** | 0.71 | 0.74 | 0.77 |
| **Lactate** | 0.70 | 0.69 | 0.64 |
| **MAP** | 0.67 | 0.61 | 0.62 |
| **HR** | 0.58 | 0.58 | 0.55 |
| **CI** | 0.76 | 0.73 | 0.61 |
| **CVP** | 0.63 | 0.55 | 0.63 |

Table S2. Area under curve (AUC) for predicting 90-day mortality of bioactive adrenomedullin (bio-ADM), lactate, mean arterial pressure (MAP), heart rate (HR), cardiac index (CI), and central venous pressure (CVP) measured at 48 to 96 hours of the detection of cardiogenic shock.
